# Mouth of the Infant at Birth

**Published:** 1891-08

**Authors:** 


### Mouth of the Infant at Birth.

The Edinburgh *Medical Journal*, November, 1890, contains a paper of unusual interest by Dr. J. W. Ballantyne, upon the anatomy of the head of new born infants, in which he gives the following description of the oral cavity:

In all the sections of the heads of infants which I have made, the mouth was seen as a potential cavity, the dorsum linguæ came into contact with the vault of the palate above, and the tongue was in apposition to the inside of the cheeks and gums laterally. In all the specimens, also, the tip of the tongue lay upon the upper surface of the lower gums. It is a fact worthy of note, that even when the mouth is tightly closed the gums do not come into contact. This fact, which is revealed by frozen sections, Symington specially dwells upon, as showing that provision exists at birth for a considerable development of the alveolar, arches and teeth before the gums of the two jaws can really meet. I have not, however, been able in my cases, to show that the distance between the jaws is so great as Symington found it, namely, six mms., for in the specimens examined, it measured from two to four mms., and in one case the jaws were in contact. Another peculiarity about the buccal cavity in the new-born infant, is the fact that the lower jaw lies in a plane posterior to that of the upper jaw. In a sagittal vertical section of the head, the anterior surface of the lower jaw is seen to be in the same vertical plane as the posterior surface of the upper jaw.

As life advances the jaws come into line with each other, and with the development of the teeth, the space between the gums disappears.

If the tip of the tongue be raised in the case of the new-born infant, two folds of the mucous membrane are seen, one of which, the larger and outer, has a dentated margin, and is called the plica fimbriata; the other, which is smaller and is situated nearer to the middle line and the frænum, is known as the plica sublingualis.

In one of my cases, there was found under the tongue, on the right side, a congenital ranula, which contained a small quantity of clear limpid fluid. H. Ranke has, in a recent paper, (Ein Sangpolster in der menschlichere Backe, *Virch. Arch.*, Bd. xcvii, p.p. 527–547) drawn special attention to pads of adipose tissue, which exist in the cheeks of new-born infants, and which are, as Symington shows, present also in the child.

Ranke was led to the study of these bodies by the fact, that in a child one year old, in a state of great emaciation from continued diarrhœa, the cheeks presented a swollen appearance. This swelling, he found to be due to the presence of a distinctly encapsulated mass of adipose tissue, the so-called sucking pad (Sangpolster).

He made sections of the face, coronal and horizontal, in the new-born infant, and also dissections from the skin surface inwards, and found that these pads were distinct structures which were not continuous with the subcutaneous adipose tissue. In several of my sections the relations of these pads could be seen, and they were always easily differentiated from the surrounding fat, from the fact, that on putting the sections into spirit, the pads changed their color slightly, and shrank from the adjacent tissues in one case to the extent of being easily removable.

Each pad lies in the neighborhood of the duct of the parotid gland, upon the buccinator, and partly upon the masseter muscle, and has superficial to it the musculus resorius of santorini. An offshoot from the pad passes into the spheno-palatine and zygomatic fossa.

Each has a vertical diameter of about 2 ctms., a transverse of about 1.5 ctm., and an antero posterior of a little over 1 ctm. They are found not only in the infant, but also in the child and adult, and are present even when the adipose tissue in other parts of the body is extremely small in amount. They are, no doubt, connected physiologically with the act of sucking, hence the name of sucking cushion given to them, and probably act by distributing equally the atmospheric pressure, and preventing the drawing inwards of the buccinator muscle between the gums during the efforts of suction when a vacuum is created in the buccal cavity.

